# Predictive Big Data Analytics using the UK Biobank Data

**DOI:** 10.1038/s41598-019-41634-y

**Published:** 2019-04-12

**Authors:** Yiwang Zhou, Lu Zhao, Nina Zhou, Yi Zhao, Simeone Marino, Tuo Wang, Hanbo Sun, Arthur W Toga, Ivo D Dinov

**Affiliations:** 1 Statistics Online Computational Resource (SOCR), Department of Health Behavior and Biological Sciences, University of Michigan, Ann Arbor, MI USA; 2 Laboratory of Neuro Imaging, USC Stevens Neuroimaging and Informatics Institute, Keck School of Medicine of USC, University of Southern California, Los Angeles, CA USA; 3 Department of Computational Medicine and Bioinformatics, University of Michigan, Ann Arbor, MI USA; 4 Michigan Institute for Data Science, University of Michigan, Ann Arbor, MI USA; 5 Department of Biostatistics, University of Michigan, Ann Arbor, MI USA; 6 Department of Statistics, University of Michigan, Ann Arbor, MI USA

## Abstract

The UK Biobank is a rich national health resource that provides enormous opportunities for international researchers to examine, model, and analyze census-like multisource healthcare data. The archive presents several challenges related to aggregation and harmonization of complex data elements, feature heterogeneity and salience, and health analytics. Using 7,614 imaging, clinical, and phenotypic features of 9,914 subjects we performed deep computed phenotyping using unsupervised clustering and derived two distinct sub-cohorts. Using parametric and nonparametric tests, we determined the top 20 most salient features contributing to the cluster separation. Our approach generated decision rules to predict the presence and progression of depression or other mental illnesses by jointly representing and modeling the significant clinical and demographic variables along with the derived salient neuroimaging features. We reported consistency and reliability measures of the derived computed phenotypes and the top salient imaging biomarkers that contributed to the unsupervised clustering. This clinical decision support system identified and utilized holistically the most critical biomarkers for predicting mental health, e.g., depression. External validation of this technique on different populations may lead to reducing healthcare expenses and improving the processes of diagnosis, forecasting, and tracking of normal and pathological aging.

## Introduction

The UK Biobank is a National Health Service data archive providing rich human health information across demographics, health, and disease. It offers incredible research opportunities for the entire worldwide scientific community^1^. The major objective of the UK Biobank is to improve the prevention, diagnosis, and treatment of a wide range of serious and life-threatening illnesses, such as cancer, heart disease, diabetes, depression, etc. Beginning in 2006, a total of 500,000 volunteers aged 40–69 are being followed for years to record their demographic, medical, and other health-related information. This is a longitudinal study with some parts of the data still being gathered. Because of the large number of participants and the long follow-up time, managing and interpreting the data in the archive presents many challenges related to the data size, format complexity, feature heterogeneity, and sampling incongruence of the observations. For instance, the UK Biobank includes irregularly-sampled longitudinal clinical, demographic, and imaging biomarkers. It is a big challenge to harmonize the data elements and extract useful information from such a complex dataset. The key objective of this paper is to interrogate the main determinants of common mental illness, especially depression, by using model-free machine-learning analytical methods.

Since the release of the UK Biobank data, researchers globally have been examining the relationships between a wide range of physical or mental illness and the available clinical, demographic, imaging, or genomic markers. One prior report^2^ studied the associations of developing obesity and related disorder with fast food and physical activity environments for mid-life adults aged 40–70 using the UK Biobank cross-sectional observational data. The authors demonstrated that greater density of physical activity facilities around home was significantly associated with smaller waist circumference, lower BMI, and reduced body fat percentage. Another prospective population-based study using the UK Biobank focused on the characterization of predictors for five-year mortality in middle-aged to elderly individuals^3^. The study examined the associations between most of the available measurements and the five-year all-cause and cause-specific mortality. The authors discovered that self-reported health, such as unable to work because of sickness or disability, was the strongest predictor of all-cause mortality in men and a previous cancer diagnosis was the strongest predictor of all-cause mortality in women. When excluding individuals with major disease or mental disorders, measures of smoking habits were the strongest predictors of all-cause mortality. Yes, smoking and other strongest predictors may simply be obtained by quick questionnaires and without extensive physical examination. Thus, for high-risk individuals some specific univariate clinical outcomes may easily be identified leading to establishing of effective public health policies. Many complex heterogeneous disorders and polymorphic and their detection, modeling, tracking and analytics require deeper computable phenotyping. A genome-wide association study of cognitive functions and educational attainment in UK Biobank participants was carried out in 2016^4^. This study investigated the genetic contributions to variation in tests of three cognitive functions and in educational attainment. It demonstrated that the genome-wide significant single-nucleotide polymorphism (SNP)-based associations were located on several specific genes: ATXN2, CYP2DG, APBA1 and CADM2. In addition, this study reported significant SNP-based heritability of 31% for verbal-numerical reasoning, 5% for memory, 11% for reaction time, and 21% for educational attainment. Although a lot of interesting findings have been discovered based on the studies of UK Biobank clinical and demographic features, few studies have used structural and functional brain neuroimaging biomarkers to examine mental health^5^. Currently, most neuroimaging studies continue to utilize modest sample sizes and limited amounts of data collected for each subject, which potentially may reduce the reproducibility and replicability of the research findings^6^. In 2014, to facilitate advanced computational neuroscientific explorations, the UK Biobank began the process of inviting back 100,000 of the original volunteers for imaging scans including brain, heart, and torso^7^. With the large number of participants, the increasing overall UK Biobank data presents both problems and opportunities. For instance, the emergence of big neuroimaging data analytics uncovers the *big confounder* and the *small effect size* issues^5^. Making meaningful interpretations and deriving valid inference using big data archives is often times tricky. Some preliminary studies are underway trying to take advantage of this deluge of big imaging data. For instance, to help convert the UK Biobank neuroimaging data into useful summary information, Alfaro-Almagro and others have developed an automated processing and QC (Quality Control) pipeline that is available for use by other researchers^7^.

This manuscript aims to address three specific UK Biobank analytic challenges.

*Challenge 1 (Feature Selection)*: Reduce the high dimensionality of the derived neuroimaging biomarkers. Presently, there are thousands of morphometric measures that are computed by parcellating the brain, modeling the boundary of each region of interest, and computing intrinsic or extrinsic morphological characteristics for each region^8–10^. Simplifying the resulting derived imaging signature vector would expose the salient features that may be highly associated with specific observed computable or clinical phenotypes.

*Challenge 2 (Data harmonization)*: Integrate the derived salient neuroimaging biomarker features with the corresponding clinical and demographic data to obtain harmonized computable data objects. The latter can be interrogated using model-based statistical methods, model-free machine learning techniques, or exploratory data analytics to examine predefined associations as well as formulate new research hypotheses^11,12^.

*Challenge 3 (Data Analytics)*: Develop a decision support system capable of supervised classification, unsupervised clustering, model-free forecasting, and prediction of clinical traits. This protocol may be used to prognosticate normal development from birth to maturation and aging, as well as to predict the trajectories of various health conditions.

## Methods

The complete data preprocessing protocol is described in Supplementary Materials. Briefly, we employed the UK Biobank archive (n = 502,627 cases and k = 4,316 features) with demographic, clinical, biological specimen, imaging, genomic, and questionnaire data elements. We used the structural magnetic resonance imaging data (sMRI) to obtain 3,297 derived neuroimaging morphometry measures of the 3D neuroanatomical integrity of the participants’ brains^8,9,13^. The complete dataset was randomly divided into a training set (n = 7,931, 80%), used for clustering and model building, and an independent testing set (n = 1,983, 20%), used for external validation.

To obtain derived computed phenotypes without a priori knowledge or specific clinically relevant traits, we relied on unsupervised machine learning methods. We split the entire population using two unsupervised clustering methods. K-means clustering and Ward’s hierarchical clustering^14–17^ were applied independently to the neuroimaging biomarkers to stratify the data into separate cohorts. Linear (multidimensional scaling, MDS, and principal component analysis, PCA)^18–20^ and non-linear (t-distributed stochastic neighbor embedding, t-SNE) dimensionality reduction methods^21–23^ were employed to project the high-dimensional data into 2D or 3D spaces. These low-dimensional Euclidean and curved manifold projection spaces illustrate the separation between the derived cohorts labels, as well as, the consistency of the computed phenotype clusters (see Supplementary Materials for more details). To pinpoint data features that may be highly predictive of specific computed phenotypes, we examined the distributional differences of the derived neuroimaging biomarkers as well as the quantitative, categorical, and clinical measures between the clusters. The most salient neuroimaging biomarkers discriminating between different clusters were identified using parametric (Student’s t) and non-parametric (Kolmogorov-Smirnov and Mann-Whitney-Wilcoxon) statistical tests. The important categorical variables related to mental disorders differently distributed between the cohorts were identified using Chi-square and Fisher’s exact tests. We harmonized and aggregated the important clinical and neuroimaging features and then jointly interrogated the entire merged data. We then constructed decision trees^11,24^ and random forests^25^ to predict specific clinical traits, like the presence of common mental disorders, using the identified salient imaging biomarkers and categorical variables related to mental disorders. The pipeline workflow for obtaining the neuroimaging biomarkers and R analytic scripts are provided in Fig. S1 in the Supplementary Materials.

## Results

### Unsupervised clustering of UK Biobank data into two separate computed phenotypes

**Figure 1:**
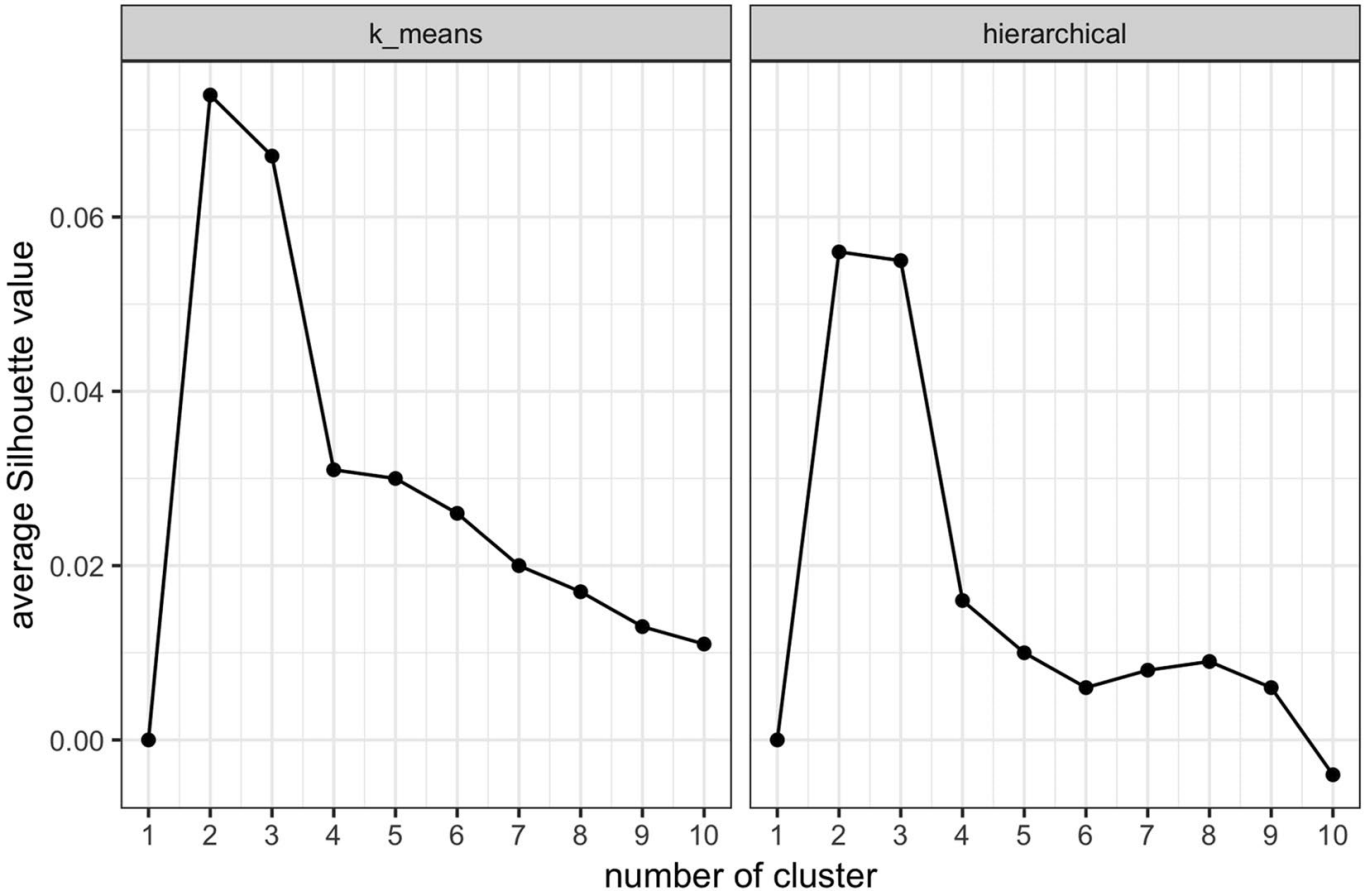
Clustering optimization based on average Silhouette value for (**a**) k-means clustering (**b**) hierarchical clustering. The optimal number of clusters is two, which maximizes the average Silhouette value for both k-means and hierarchical clustering.

The first step of unsupervised clustering is to determine the optimal number of clusters. Figure 1 shows a plot of the average silhouette value (indicator of cluster robustness and reliability) for different number of clusters. The cluster number optimization results based on total within-cluster sum of squares is shown in Fig. S2 in the Supplementary Materials. The plots of the silhouette values for both k-means clustering and hierarchical clustering suggest that the optimal number of clusters is two.

We assessed the reliability and reproducibility of the derived computed clustering phenotypes using several alternative strategies: (1) evaluate the robustness of clustering with repeated experiments, (2) validate the clustering results with illustrations based on dimensionality reduction methods, (3) compare clustering consistency between independent clustering methods, and (4) validate the computed clustering phenotypes by supervised clustering methods. To evaluate the clustering robustness, we performed 1,000 times repeated k-means clustering. The consistency among these 1,000 randomly initialized k-means clustering results is summarized in Supplementary Materials Table S1. Consistency was defined as the probability of an arbitrary pair of two subjects being clustered in the same derived computed phenotype across different clustering experiments. Based solely on the neuroimaging biomarkers, these results suggest that k-means clustering represents highly robust and consistent mapping of computed phenotypes.

**Figure 2:**
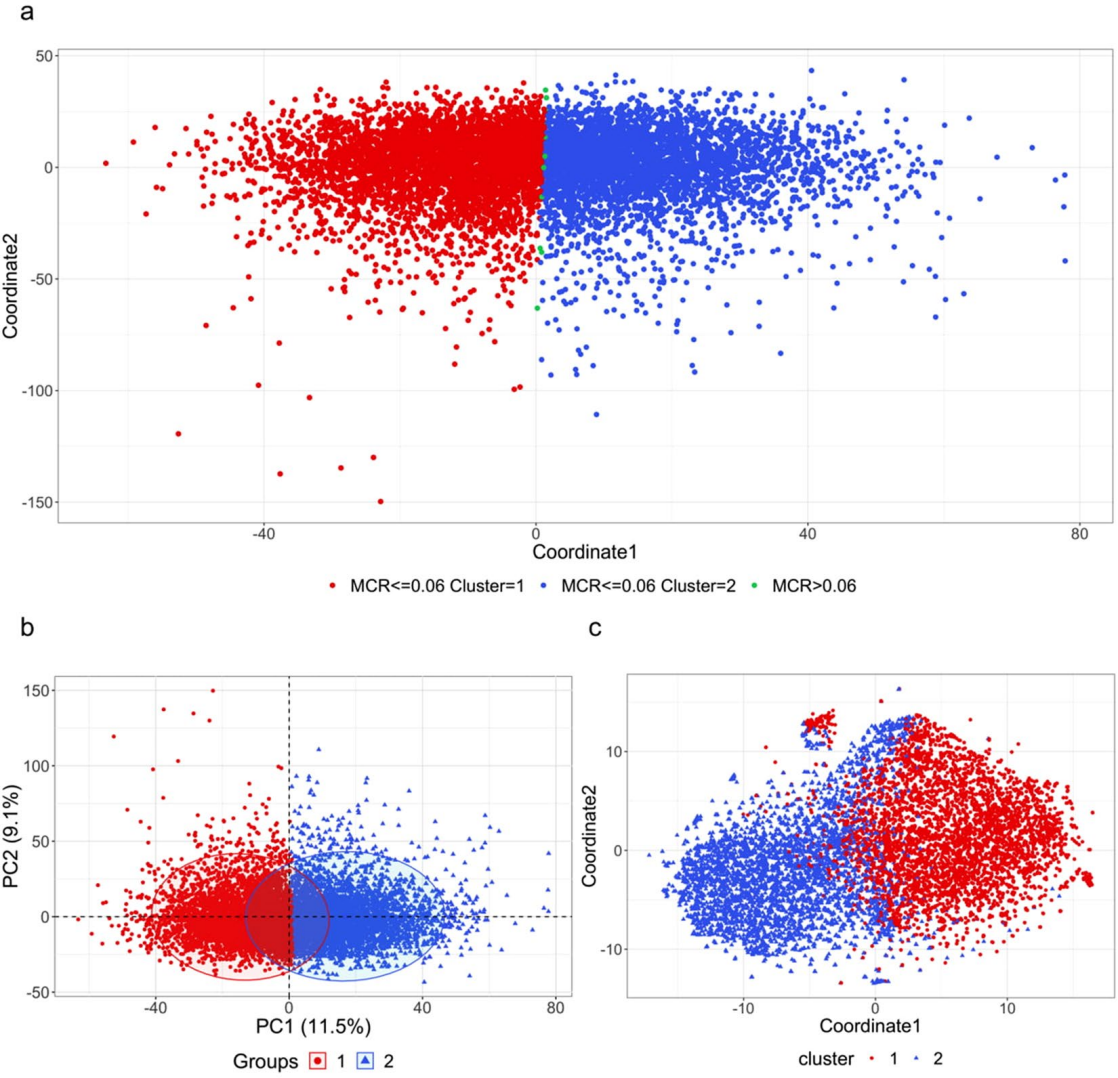
Panel a: Multidimensional scaling (MDS) for neuroimaging biomarkers with clustering labels generated by k-means clustering. MCR is the misclassification rate based on the 1,000 k-means clustering experiments. Panels b and c: 2-dimensional plots of (**b**) PCA and (**c**) t-SNE for the brain neuroimaging biomarkers with the clustering label generated by k-means clustering.

**Figure 3:**
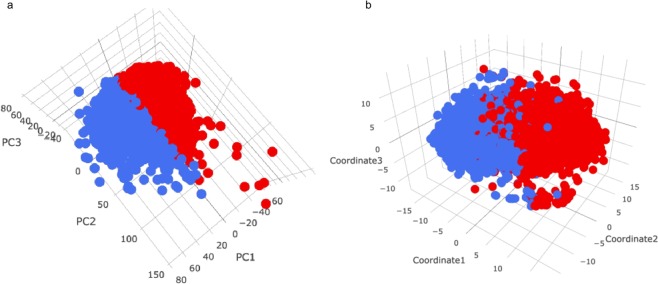
3-dimensional plots of (**a**) PCA and (**b**) t-SNE for the brain neuroimaging biomarkers with the red (cluster1) and blue (cluster2) clustering labels generated by k-means clustering.

K-means clustering of the neuroimaging biomarkers suggests the existance of structural patterns in the data. Figure 2a shows the multidimensional scaling (MDS) plot of the biomarkers color coded by the derived computed phenotypes generated by k-means clustering. The misclassification rate (MCR) is calculated based on the 1,000 k-means clustering trials as the probability a single subject being clustered into a different computed phenotype relative to its major clustering label. The subjects with higher MCR are located along the boundary of the two clusters, which is to be expected as the subjects near the boundary are more likely to be misclassified. According to Fig. 2a, we can see that although the two clusters may not be widely separated, k-means clustering generates a clear boundary between the two computed phenotypes. It appears as if the first MDS coordinate plays the most important role in this 2D separation. To further validate the clustering results using dimensionality reduction methods, 2D and 3D PCA and t-SNE plots were generated. These Euclidean and curved-manifold projections of the high-dimensional data into low-dimensional spaces provide independent evidence of the separation of the derived computed phenotypes (Fig. 2b,c and Fig. 3). Figure 2b shows that PCA yields a similar separation of clusters as MDS. Although there is no distinct separation of the two clusters, there is a clear boundary between them. Again, the first dimension plays the most important role in cluster separation. The t-SNE plot tells a similar story; however, it generates a more detailed (perhaps non-linear) structure of cluster separation (Fig. 2c). The 3D PCA and t-SNE plots show a similar separation boundary. These findings suggest that k-means clustering picks up important information from the thousands of neuroimaging biomarkers as it splits the data into two computed phenotypes.

The robustness of k-means clustering is evaluated by comparing the k-means derived computed phenotypes to those obtained by an independent hierarchical clustering. The consistency of the two data partitioning schemes is shown in Supplementary Materials Table S2. K-means and hierarchical clustering generate very consistent sub-cohort divisions. About 81.5% of the subjects are clustered into the same sub-cohort groups by both k-means and hierarchical clustering. Because of this strong clustering agreement, we focused all subsequent analyses on the results based on k-means clustering.

In addition to hierarchical clustering, the computed phenotypes derived from k-means clustering were also validated by supervised clustering methods, including k-nearest neighbor (kNN) and artificial neural network (ANN). 5-fold cross validation was applied to evaluate the consistency of the classification results. Our results showed that kNN gave a 93.7% consistency and ANN gave a 97.3% consistency of labeling the computed phenotypes derived by k-means clustering, which indicates a very strong agreement of these independent clustering and classification methods.

### Challenge 1 (feature selection): reduce the high dimensionality of neuroimaging biomarkers

**Figure 4:**
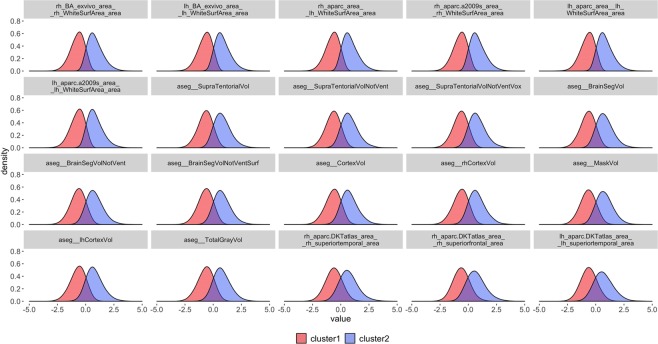
Density plots of the scaled top twenty brain neuroimaging biomarkers with the clustering label generated by k-means clustering. Details about the specific FreeSurfer^8^ derivation and interpretation of the neuroimaging biomarkers listed in Table 1 and shown in Fig. 4 are available online at https://surfer.nmr.mgh.harvard.edu/fswiki/FsTutorial/AnatomicalROI and https://surfer.nmr.mgh.harvard.edu/fswiki/FsTutorial/AnatomicalROI/FreeSurferColorLUT.

To address the first challenge of feature selection, we identified the top twenty salient biomarkers that are significantly different between the derived two computed phenotypes based on parametric and nonparametric tests comparing their distributions. The density plots of the selected twenty biomarkers are illustrated in Fig. 4. All the selected biomarkers appear fairly normally distributed, with cluster 1 having negative means and cluster 2 having positive means. This indicates that these salient biomarkers present strong signals separating the two computed phenotypes. Table 1 summarizes the descriptive statistics of the raw (unscaled) values of these biomarkers. Next, we focus the analysis on these twenty salient biomarkers.

**Table 1:** Summary statistics of the unscaled values for the top twenty brain neuroimaging biomarkers separating cluster 1 and 2.

Name	Computed Phenotype 1 (cluster 1)			Computed Phenotype 2 (cluster 2)			Significance
	Mean	Median	SD	Mean	Median	SD	
rh_BA_exvivo_area__rh_WhiteSurfArea_area	83,840	84,354	4,687	96,861	95,814	5,283	***
lh_BA_exvivo_area__lh_WhiteSurfArea_area	83,571	84,190	4,685	96,467	95,423	5,260	***
rh_aparc_area__rh_WhiteSurfArea_area	78,686	79,209	4,556	91,279	90,232	5,126	***
rh_aparc.a2009s_area__rh_WhiteSurfArea_area	78,705	79,231	4,556	91,299	90,238	5,125	***
lh_aparc_area__lh_WhiteSurfArea_area	78,418	79,022	4,545	90,870	89,864	5,103	***
lh_aparc.a2009s_area__lh_WhiteSurfArea_area	78,437	79,037	4,545	90,891	89,890	5,103	***
aseg__SupraTentorialVol	944,195	948,603	62,451	1,102,286	1,093,828	72,318	***
aseg__SupraTentorialVolNotVent	921,038	925,358	61,368	1,072,067	1,063,706	70,466	***
aseg__SupraTentorialVolNotVentVox	918,633	922,839	61,275	1,069,254	1,060,989	70,261	***
aseg__BrainSegVol	1,077,598	1,082,266	69,154	1,247,680	1,238,569	79,105	***
aseg__BrainSegVolNotVent	1,050,639	1,055,186	67,942	1,213,003	1,204,173	77,138	***
aseg__BrainSegVolNotVentSurf	1,050,038	1,054,468	67,912	1,212,341	1,203,650	77,144	***
aseg__CortexVol	431,767	433,836	28,159	496,015	492,066	31,309	***
aseg__rhCortexVol	216,033	217,094	14,119	248,268	246,293	15,757	***
aseg__MaskVol	1,479,307	1,482,621	97,626	1,700,818	1,691,706	107,502	***
aseg__lhCortexVol	215,734	216,893	14,237	247,747	245,918	15,738	***
aseg__TotalGrayVol	590,111	592,534	36,298	669,907	665,861	40,069	***
rh_aparc.DKTatlas_area__rh_superiortemporal_area	4,411	4,414	329	5,038	5,005	385	***
rh_aparc.DKTatlas_area__rh_superiorfrontal_area	8,055	8,034	751	9,475	9,382	887	***
lh_aparc.DKTatlas_area__lh_superiortemporal_area	4,723	4,716	387	5,459	5,411	472	***

Significance code: ***p-value <1 × 10^−8^. The p-values were calculated based on Whitney-Wilcoxon tests.

### Challenge 2 (data harmonization): integrate salient imaging biomarkers and clinical data

**Figure 5:**
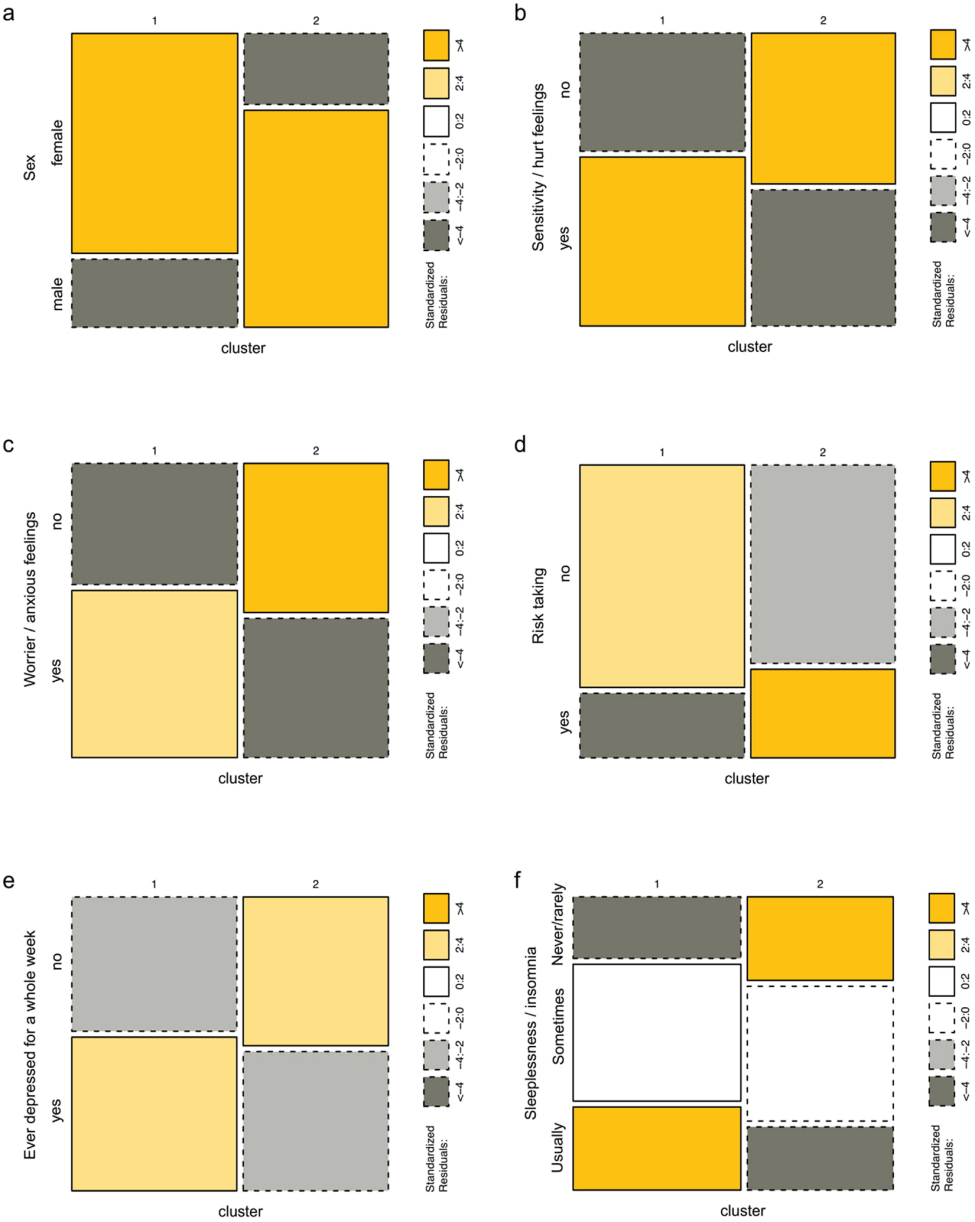
Mosaic plots for some of the significantly different categorical features detected by Chi-square test and Fisher’s exact test. The six parts of the figure include (**a**) Sex; (**b**) Sensitivity/hurt feelings; (**c**) Worrier/anxious feelings; (**d**) Risk taking; (**e**) Ever depressed for a whole week; and (**f**) Sleeplessness/insomnia. The standard residuals, reported in the right margins, indicate the significance of the differences.

To address the second challenge of data harmonization, we integrate the derived salient neuroimaging biomarkers with some clinical and demographic data and obtained a harmonized computable data object. The categorical variables that are significantly different between the two clusters, based on Chi-square tests and Fisher’s exact tests, are summarized in Supplementary Materials Table S4. The mosaic plots illustrated in Fig. 5 show distribution differences of some of the most significantly different categorical variables between the two clusters. We can see that the distributions of females and males are vastly different between the two clusters, with cluster 1 including the majority of the females and cluster 2 containing predominantly males. The other five mosaic plots show that the subjects in cluster 1 tend to (1) have more sensitive/hurt feelings and more worried/anxious feelings; (2) have less willingness to take risks; (3) are more likely to feel depressed for a whole week; and (4) have more difficulties in sleeping. All these results may be highly associated with the significant gender disparity between the clusters. Albeit these findings illustrate how unsupervised clustering may be used for exploratory as well as confirmatory analytics in mental disorders, the same approach may be used to tackle different types of health conditions or to monitor normal development and aging.

### Challenge 3 (data analytics): develop a decision support system using random forests

Once we identified the salient neuroimaging biomarkers and categorical variables, we proceeded to examine the data in its entirety, e.g., look for potential associations between the categorical variables related to mental illness and the neuroimaging biomarkers. In order to address the third challenge (i.e., developing a decision support system capable of predicting the disease state), we started by fitting a single decision tree. Supplementary Materials Figure S3 illustrates a direct example of an explicit decision support system that can be used for prediction of the mental state, e.g., sensitivity/hurt feelings. This specific decision tree shows that three of the salient biomarkers are important in the decision classification of the participants, namely *rh_aparc.DKTatlas_area__rh_superiorfrontal_area*, *lh_aparc.DKTatlas_area__lh_superiortemporal_area*, and *aseg__MaskVol*. The other two features are both categorical variables and include “*Worry too long after embarrassment*,” which plays the most important role in the classification. Subjects responding “yes” to the question “*Worry too long after embarrassment*” are more likely to have sensitivity/hurt feelings than those responding “*no*.” Using the remaining variable together with the three neuroimaging biomarkers provides a deeper classification phenotyping. For instance, subjects with worrier/anxious feelings and *lh_aparc.DKTatlas_area__lh_superiortemporal_area* larger than −1.158 (scaled values) are 16.2% less likely to *have sensitivity/hurt feelings*. Similarly, subjects without worrier/anxious feelings and *lh_aparc.DKTatlas_area__lh_superiortemporal_area* larger than −1.393 are 26.7% less likely to *have sensitivity/hurt feeling*s. Supplementary Materials Figure S3 illustrates how the decision tree can be used as a clinical decision support system guiding physicians in using the specific imaging biomarkers and categorical variables for prognostication or treatment planning. In addition, this information may be useful to guide prospective studies, i.e., what prospective data should be collected (for future clinical trials) or mined (for retrospective data analytics). Of course, this simple example is just an illustration. To avoid the difficulties of unavoidable large variance or large bias issues in using single decision trees, in practice, we use the much more reliable ensemble classification and regression methods like random forests.

**Figure 6:**
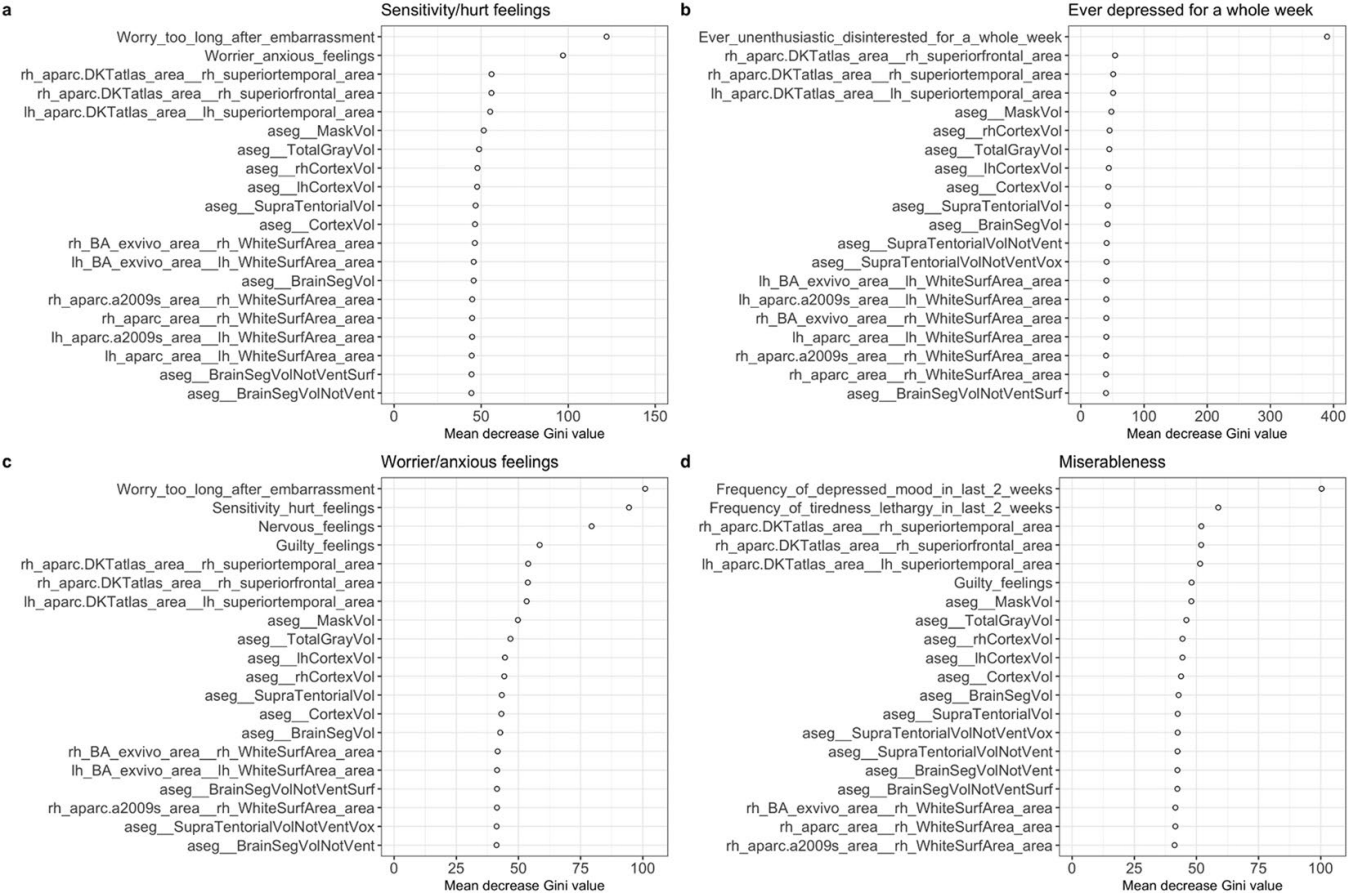
Variable importance plots for four different outcome predictions: (**a**) *sensitivity/hurt feelings*; (**b**) *ever depressed for a whole week*; (**c**) *worrier/anxious feelings*; and (**d**) *miserableness* based on mean decrease Gini values by random forest.

Next, we focus on developing a classifier that can predict the presence of some specific mental disorders. Random forest prediction relies on boosting hundreds of decision tree classifiers using the combination of the salient neuroimaging biomarkers and the salient categorical features. Figure 6 illustrates four examples of the top twenty variables identified to be important in the prediction of “*sensitivity/hurt feelings*,” “*ever depressed for a whole week*,” “*worrier/anxious feelings*,” and “*miserableness*” based on the mean decrease of the Gini values. “*Worry too long after embarrassment*,” “*worrier/anxious feelings*,” and 18 other neuroimaging biomarkers are listed as the top twenty features for predicting “*sensitivity/hurt feelings*.” In developing the decision rules for “*ever depressed for a whole week*,” we first included all selected imaging biomarkers and all categorical features. A deeper examination into the categorical variables revealed that two variables, “*seen doctor (GP) for nerves, anxiety, tension or depression*” and “*frequency of depressed mood in last 2 weeks*,” were highly associated with the response variable we predicted. Therefore, we retrained the random forest classifier excluding these two specific predictors to avoid confounding problems. The result showed that “*Ever unenthusiastic/disinterested for a whole week*” was the most important predictor in forecasting depression. The other features had approximately similar contributions. Indeed, depression, and other mental health disorders, represent complex heterogeneous conditions, and one would not expect a small number of imaging biomarkers to yield extremely accurate, consistent, or reliable predictions. In the prediction of “*worrier/anxious feelings*” and “*miserableness*,” the salient neuroimaging biomarkers also played an important role. Table 2 summarizes the cross-validated random forest prediction accuracy (with 95% confidence intervals, CI), sensitivity and specificity for predicting four specific mental health outcomes: “*sensitivity/hurt feelings*,” “*ever depressed for a whole week*,” “*worrier/anxious feelings*,” and “*miserableness*.” The consistent 70–80% accuracy across these four mental conditions suggests that these machine-learning strategies may be useful to support physicians in their diagnosis, prognosis, and disease progression tracking. The prediction performance of the established models on the testing dataset is summarized in Table 3, which indicates a high prediction consistency for an independent dataset.

**Table 2:** Cross-validated random forest prediction results for “*sensitivity/hurt feelings*,” “*ever depressed for a whole week*,” “*worrier/anxious feelings*,” and “*miserableness*.”

	Accuracy	95% CI of Accuracy	Sensitivity	Specificity
Sensitivity/hurt feelings	0.720	(0.686, 0.753)	0.684	0.754
Ever depressed for a whole week	0.778	(0.746, 0.807)	0.912	0.640
Worrier/anxious feelings	0.739	(0.706, 0.771)	0.723	0.755
Miserableness	0.743	(0.710, 0.775)	0.867	0.550

**Table 3:** Random forest prediction results for “*sensitivity/hurt feelings*,” “*ever depressed for a whole week*,” “*worrier/anxious feelings*,” and “*miserableness*” in the testing dataset.

	Accuracy	95% CI of Accuracy	Sensitivity	Specificity
Sensitivity/hurt feelings	0.708	(0.677, 0.737)	0.690	0.724
Ever depressed for a whole week	0.773	(0.745, 0.800)	0.908	0.624
Worrier/anxious feelings	0.725	(0.695, 0.754)	0.735	0.716
Miserableness	0.747	(0.718, 0.775)	0.880	0.521

## Discussion

The UK Biobank is a complex data archive with an ongoing data collection process. The large number of observations and the complex composition of the data elements make it very difficult for researchers to statistically mine and computationally generate precise, reliable and consistent inference. In this manuscript, we employ a three-challenge approach to extract useful information from the UK Biobank, like deriving prediction models detecting the presence and tracking the progression of depression and other mental illness. The first challenge was to reduce the dimension of the dataset and identify salient features among thousands of the variables. By performing unsupervised clustering, we successfully computed derived phenotypes (clusters) that were consistent (across methods) and reliable (across experiments). We also identified the top twenty salient imaging biomarkers that contribute most to the separation of the two clusters. Examining the distributions of these twenty neuroimaging biomarkers, we found that they appear to be approximately normally distributed, with cluster 1 having predominantly negative means and cluster 2 having positive means. We tested many more of the neuroimaging biomarkers to determine whether they were significantly differently distributed between the two clusters. Figure S4, in Supplementary Materials section, shows the distributions of twenty neuroimaging biomarkers that ranked in the middle, and another twenty biomarkers that ranked at the bottom of feature significance according to parametric and nonparametric tests. These groups of features were not significantly different across the computed phenotypes and their density plots illustrate no obvious differences. Therefore, our forecasting and prediction of mental health outcomes only used the top twenty imaging biomarkers. The number of selected imaging biomarkers was determined by the consistency of their significances in separating the computed phenotypes based on k-means clustering and hierarchical clustering. All the top twenty selected neuroimaging biomarkers were common according to the rankings of the significance tests with clustering results generated by k-means and the hierarchical clustering.

Challenge two involved harmonizing and aggregating imaging, clinical and demographic data elements and the joint interrogation of the holistic dataset. We demonstrated how unsupervised clustering may be used for either exploratory or confirmatory analytics in many health studies.

The final challenge addressed in this study was to develop an effective decision support system that is capable of detecting the presence of and predicting the progression of common illnesses. Our approach relies on unsupervised learning of derived neuroimaging biomarkers and categorical phenotypic features. Following the unsupervised clustering, we performed Chi-square and Fisher’s exact tests to determine the categorical variables that are discriminating between the two clusters. One interesting discovery from the tests of the categorical variables is the significant gender disparity between the clusters. This supports previous reports of association between the gender disparity and the prevalence of mental disorders^26–28^. The subjects in the majority female cluster were more likely to experience mental disorders. This finding suggests gender differences in mental health. It is demonstrated that across many nations, cultures, and ethnicities, females are about twice as likely as males to develop depression^29,30^. Women have a lifetime prevalence for major depressive disorder of 21.3%, compared with 12.7% in men^31^. In addition to depression, females are more likely to express anxiety and worry, and also reported a more negative problem orientation and engaging in more thought suppression than males^32,33^. Our finding is consistent with the previous discoveries, indicating that females are more vulnerable to emotional fluctuation and mental disorders.

Aggregating the salient neuroimaging biomarkers and the selected categorical variables allowed us to generate a computable data object that can be interrogated to examine predefined associations (confirmatory analytics) as well as formulate new research hypotheses (exploratory analytics). The final decision guidelines for predicting some mental disorders, e.g., depression, were developed using a random forest classifier. Despite the fact that there are similarities between random forest and individual decision tree classification, the decision-making criteria determined by random forest prediction cannot be directly explicated the way a single decision tree classification can be explained. As our decision tree classifier demonstrated, categorical variables seem to play a dominant role in the classification. However, involvement of the neuroimaging biomarkers can provide additional stratification complementing the classification procedure. Random forest classification ranked some imaging biomarkers higher than some of the categorical variables, which illustrates differences with the single decision tree classifier. In the Supplementary Materials section, we also demonstrate an approach to derive deeper computable phenotypes by stratifying clusters within the low-dimensional t-SNE manifold (see Supplementary Materials Figures S5 and S6, as well as Tables S4 and S5). Figure S6b and Table S6 show how biomarkers representing the size of specific cortical surface areas in the right hemisphere might indicate reduced functional activities, as many prior studies have shown. For instance, Kuperberg and colleagues demonstrated selective thinning of the cerebral prefrontal cortices (including precentral and postcentral gyri) in patients with schizophrenia^34^. Others have shown similar reductions of pre- and post-central areas in bipolar disease and Williams syndrome^35–37^.

The observed consistency of the derived computed phenotypes and the reliability of the chosen top salient biomarkers contributing to the unsupervised clustering suggest that the information structure in the UKBB dataset can be exploited using various analytical techniques. The example of a rudimentary clinical decision support system we illustrated here specifically identified the critical biomarkers used in forecasting of depression, anxiety, and mood disorders.

As firm supporters of open-science, the authors encourage independent validation, reproducibility and expansion of the reported results. Innovative collaborations using similar techniques may reduce healthcare costs and improve patient diagnosis and disease tracking of normal and pathological conditions. The entire computational protocol, software code, pipeline workflows, and R-scripts are available on the SOCR GitHub site (https://github.com/SOCR/UKBB_Analytics). All UK Biobank data is available online at http://www.ukbiobank.ac.uk.

## Supplementary information

Supplementary Materials (Appendix)

## References

[1] Palmer LJ (2007). UK Biobank: bank on it. The Lancet.

[2] Mason KE, Pearce N, Cummins S (2018). Associations between fast food and physical activity environments and adiposity in mid-life: cross-sectional, observational evidence from UK Biobank. *Lancet*. Public Health.

[3] Ganna A, Ingelsson E (2015). 5 year mortality predictors in 498,103 UK Biobank participants: a prospective population-based study. Lancet.

[4] Davies G (2016). Genome-wide association study of cognitive functions and educational attainment in UK Biobank (N = 112 151). Mol Psychiatry.

[5] Alfaro-Almagro F (2018). Image processing and Quality Control for the first 10,000 brain imaging datasets from UK Biobank. NeuroImage.

[6] Smith SM, Nichols TE (2018). Statistical Challenges in “Big Data” Human Neuroimaging. Neuron.

[7] Alfaro-Almagro F (2018). Image processing and Quality Control for the first 10,000 brain imaging datasets from UK Biobank. Neuroimage.

[8] Fischl B (2012). FreeSurfer. Neuroimage.

[9] Tu Z (2008). Brain Anatomical Structure Segmentation by Hybrid Discriminative/Generative Models. IEEE Transactions on Medical Imaging.

[10] Dinov I (2016). Predictive Big Data Analytics: A Study of Parkinson’s Disease using Large, Complex, Heterogeneous, Incongruent, Multi-source and Incomplete Observations. PLoS One.

[11] Dinov, I. *Data Science and Predictive Analytics: Biomedical and Health Applications using R*. http://Predictive.Space (Springer International Publishing, 2018).

[12] Dinov I (2016). Methodological Challenges and Analytic Opportunities for Modeling and Interpreting Big Healthcare Data. GigaScience.

[13] Dinov I (2010). Neuroimaging Study Designs, Computational Analyses and Data Provenance Using the LONI Pipeline. Plos One.

[14] Almeida JS, Prieto CA (2013). Automated unsupervised classification of the Sloan Digital Sky Survey stellar spectra using k-means clustering. The Astrophysical Journal.

[15] Aggarwal, C. C. & Reddy, C. K. *Data clustering: algorithms and applications* (CRC Press, 2013).

[16] Filzmoser P, Baumgartner R, Moser E (1999). A hierarchical clustering method for analyzing functional MR images. Magnetic Resonance Imaging.

[17] Mirkin, B. In *Classification, data analysis, and data highways* 172–181 (Springer, 1998).

[18] Steyvers, M. Multidimensional scaling. *Encyclopedia of cognitive science* (2002).

[19] Jolliffe, I. *Principal component analysis* (Wiley Online Library, 2002).

[20] Murtagh F (1983). A survey of recent advances in hierarchical clustering algorithms. The Computer Journal.

[21] Van Der Maaten L (2014). Accelerating t-SNE using tree-based algorithms. Journal of machine learning research.

[22] Maaten LVD, Hinton G (2008). Visualizing data using t-SNE. Journal of machine learning research.

[23] Van Erven T, Harremos P (2014). Rényi divergence and Kullback-Leibler divergence. IEEE Transactions on Information Theory.

[24] Twala B, Jones M, Hand DJ (2008). Good methods for coping with missing data in decision trees. Pattern Recognition Letters.

[25] Breiman L (2001). Random forests. Machine learning.

[26] Salk RH, Hyde JS, Abramson LY (2017). Gender differences in depression in representative national samples: Meta-analyses of diagnoses and symptoms. Psychological bulletin.

[27] Yu S (2018). Uncovering the hidden impacts of inequality on mental health: a global study. Translational psychiatry.

[28] Lee Y-C (2017). Cost of high prevalence mental disorders: Findings from the 2007 Australian National Survey of Mental Health and Wellbeing. Australian & New Zealand Journal of Psychiatry.

[29] Weissman MM (1996). Cross-national epidemiology of major depression and bipolar disorder. JAMA.

[30] Nolen-Hoeksema, S. Sex differences in depression. *Standard, CA: Standard University Press* (1990).

[31] Kessler RC, McGonagle KA, Swartz M, Blazer DG, Nelson CB (1993). Sex and depression in the National Comorbidity Survey. I: Lifetime prevalence, chronicity and recurrence. J Affect Disord.

[32] Simonds VM, Whiffen VE (2003). Are gender differences in depression explained by gender differences in co-morbid anxiety?. J Affect Disord.

[33] Robichaud M, Dugas MJ, Conway M (2003). Gender differences in worry and associated cognitive-behavioral variables. J Anxiety Disord.

[34] Kuperberg GR (2003). Regionally localized thinning of the cerebral cortex in schizophrenia. Archives of general psychiatry.

[35] Lemaitre H (2012). Normal age-related brain morphometric changes: nonuniformity across cortical thickness, surface area and gray matter volume?. Neurobiology of aging.

[36] Rimol LM (2012). Cortical volume, surface area, and thickness in schizophrenia and bipolar disorder. Biological psychiatry.

[37] Thompson PM (2005). Abnormal cortical complexity and thickness profiles mapped in Williams syndrome. Journal of Neuroscience.

