# Supplementary material for: Predictive Big Data Analytics using the UK Biobank Data
Source: Sci Rep. 2019 Apr 12;9:6012. doi: 10.1038/s41598-019-41634-y (PMC6461626; doi:10.1038/s41598-019-41634-y)
Supplement: Supplementary file 1 — Supplementary Materials (Appendix) [file 41598_2019_41634_MOESM1_ESM.docx]

**Supplementary Materials to the article**

**Predictive Big Data Analytics**

**using the UK Biobank Data**

Yiwang Zhou ^1,3,5^, Lu Zhao ^2^, Nina Zhou ^1,3,5^, Yi Zhao ^1^, Simeone Marino ^1^,

Tuo Wang ^1,6^, Hanbo Sun ^1,6^, Arthur Toga ^2^, Ivo Dinov ^1,2,3,4^

^1^ Statistics Online Computational Resource (SOCR),

Department of Health Behavior and Biological Sciences,

University of Michigan, Ann Arbor, MI, USA

^2^ Laboratory of Neuro Imaging, USC Stevens Neuroimaging and Informatics Institute,

Keck School of Medicine of USC, University of Southern California, Los Angeles, CA 90033

^3^ Department of Computational Medicine and Bioinformatics,

^4^ Michigan Institute for Data Science,

^5^ Department of Biostatistics,

^6^ Department of Statistics,

University of Michigan, Ann Arbor, MI, USA

**Correspondence**:

Ivo D. Dinov, PhD

Director, Statistics Online Computational Resource

Department of Health Behavior and Biological Sciences

University of Michigan

Ann Arbor, MI 48109

[statistics@umich.edu](mailto:statistics@umich.edu)

[www.umich.edu/~dinov](http://www.umich.edu/~dinov)

## **Methods**

### *1. Data preparation.*

The UK Biobank raw dataset contains a total of 502,627 cases and 4,316 variables, including demographic features, clinical measurements, biological sample data, imaging data, genomic data, interview answers, etc. The UK Biobank cases that have brain neuroimaging scans, which are used to obtain the derived imaging biomarkers, include a total of 9,914 volunteers and 3,297 biomarkers. Only about 11,000 participants had structural magnetic resonance imaging scans, and some scans failed quality control (QC) or automated volume parcellations. Integration of the clinical with the derived neuroimaging biomarkers data yielded a total of 9,914 observations and 7,613 variables. The clinical and phenotypic data included many incomplete cases with different degrees of missingness, whereas the neuroimaging biomarkers represent a fully complete data. The complete data is divided into a training dataset (n=7,931, 80%), used for clustering and model building, and a testing dataset (n=1,983, 20%), used for validation.

We designed an end-to-end complete data analytic workflow, which is summarized in **Figure S1**. After the integration of the UK Biobank raw dataset and the derived brain neuroimaging biomarkers, unsupervised clustering is performed solely based on the imaging signature vectors. The identified top twenty imaging biomarkers represent the most salient features discriminating between different clusters. The significance of their impact is quantifies using parametric (Student’s t-test) and non-parametric (Kolmogorov-Smirnov test and Mann-Whitney-Wilcoxon) tests. The selected significant neuroimaging biomarkers together with the machine-learning based clustering labels (computed phenotypes) are then used to examine the distributions of the categorical features in the data. Salient and unimportant categorical features are identified using Chi-square and Fisher’s exact tests. Finally, we used jointly the clinical data, demographic features, and derived neuroimaging biomarkers to build decision rules, which enabled the prediction of the presence and progression of common mental disorders using random forests.


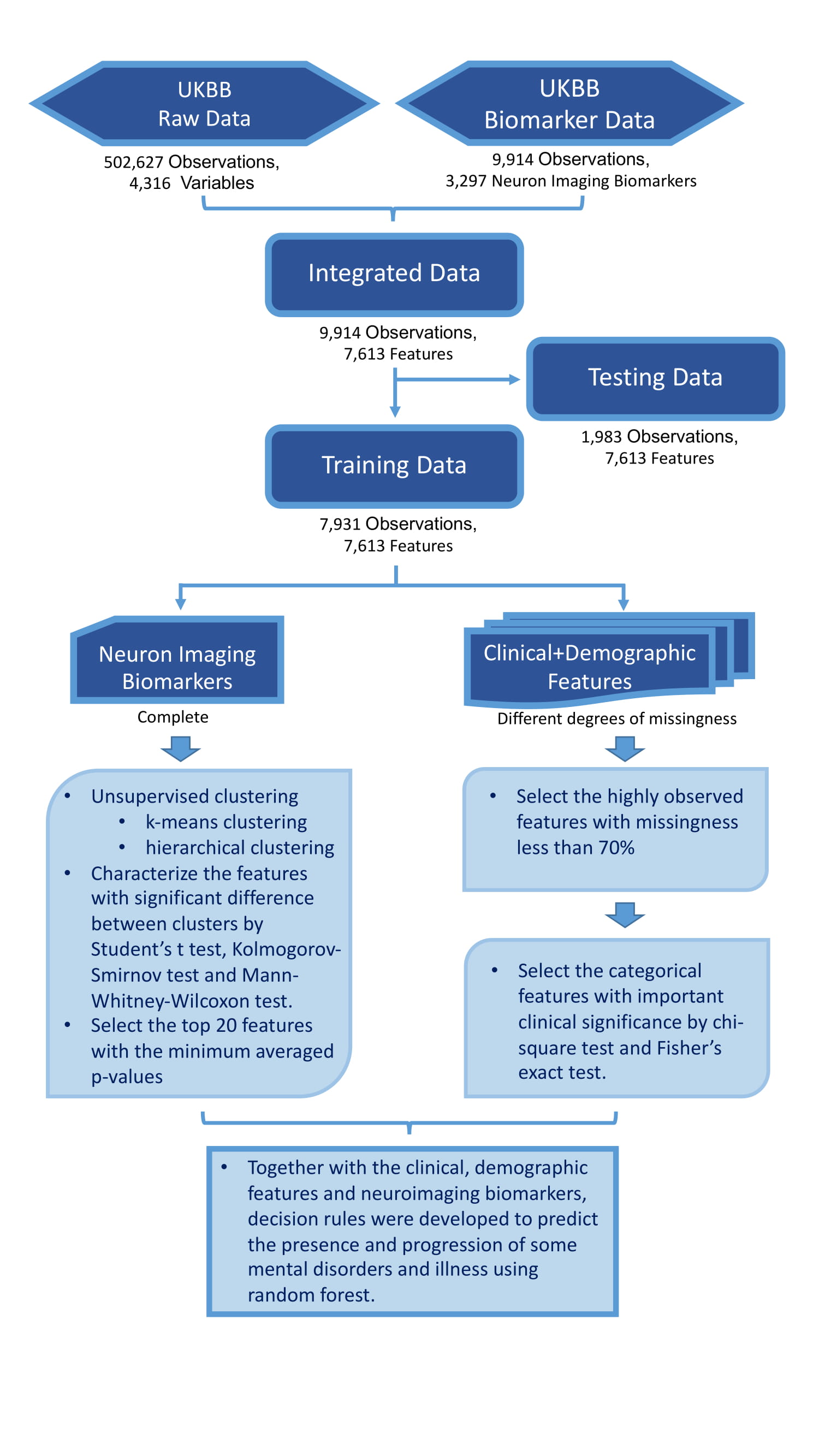


**Figure S1**. Data Analysis workflow.

### **2. Unsupervised clustering**

*2.1 K-means clustering*

The k-means algorithm is one of the most commonly used algorithms for unsupervised clustering [^1^](#_ENREF_1)^,^[^2^](#_ENREF_2). K-means clustering aims to partition $n$ observations into $k$ clusters in which each observation belongs to the cluster with nearest mean, serving as a prototype of the cluster. The algorithm works as follows: 1) Randomly select $k$ points as cluster centers. 2) Determine the maximum extent of the cluster boundaries that all have maximal distance from their clusters. The assignment of each point to a certain cluster is based on computing the least within-cluster sum of squares according to the chosen distance. 3) Update the center of the cluster to new means of the cluster centroid locations. The optimal number of cluster $k$ is selected according to silhouette value and the total within-cluster sum of squares. The silhouette value measures the similarity (cohesion) of a data point to its cluster relative to other clusters. High silhouette value suggests that the data matches its own cluster well. A clustering algorithm performs well when most silhouette values are large. Poor clustering may imply that the algorithm configuration may have too many or too few clusters. Suppose a clustering method groups all data points $\left\{ X_{i} \right\}, i=1,\ldots,n$ into $k$ clusters and define:

1. $d_{i}$ as the average dissimilarity of $X_{i}$ with all other data points within its cluster. $d_{i}$ captures the quality of the assignment of $X_{i}$ to its current class label. Smaller or larger $d_{i}$ values suggest better or worse overall assignment for $X_{i}$ to its cluster, respectively. The average dissimilarity of $X_{i}$ to a cluster $C$ is the average distance between $X_{i}$ and all points in the cluster of points labeled as $C$.
2. $l_{i}$ as the lowest average dissimilarity of $X_{i}$ to any other cluster, that $X_{i}$ is not a member of. The clustering corresponding to $l_{i}$, the lowest average dissimilarity, is called the $X_{i}$ neighboring cluster, as it is the next best fit cluster for $X_{i}$.

Then, the silhouette value of $X_{i}$ is defined by:

$-1\leq s_{i}=\frac{l_{i}-d_{i}}{max\{l_{i},d_{i}\}}\equiv\left\{ \begin{aligned} 1-\frac{d_{i}}{l_{i}}, if d_{i}<l_{i} \\ 0, if d_{i}=l_{i}\leq1 \\ \frac{l_{i}}{d_{i}}-1, if d_{i}>l_{i} \end{aligned} \right.$.

*2.2 Hierarchical clustering (HC)*

Hierarchical clustering is a method of cluster analysis which seeks to build a hierarchy of clusters [^3^](#_ENREF_3). There are two major types of hierarchical clustering, agglomerative clustering and divisive clustering [^4^](#_ENREF_4). Agglomerative clustering treats each observation as its own cluster, and pairs of clusters are merged as one according to their similarities. Divisive clustering starts with all observations in one cluster, and then splits are performed recursively according to the dissimilarities. In order to decide which clusters should be combined (for agglomerative clustering), or where a cluster should be split (for divisive clustering), a measure of dissimilarities between sets of observations is required. Most hierarchical clustering approaches approximate some distance measure and use a linkage criterion which specifies the dissimilarity of sets as a function of the pairwise distance of observations in the sets. In this study, we employed Euclidean distance measure in the hierarchical clustering. There are four major types of linkage criteria, complete-linkage criterion, single-linkage criterion, average linkage criterion, and Ward’s criterion [^5^](#_ENREF_5). The optimal linkage criterion is selected according to the agglomerative coefficient, which describes the strength of the clustering structure that has been obtained by group average linkage. We used Ward linkage criterion according to the agglomerative coefficient. In our analysis, the optimal number of clusters, $k,$ is also determined according to silhouette value and the total within-cluster sum of squares.

**3. Supervised classification**

*3.1 K-nearest neighbors classification (kNN)*

K-nearest neighbors algorithm is a non-parametric method that can be used for both classification and regression. When kNN is applied for classification, the training dataset contains observations in the multidimensional feature space with known labels. An unlabeled observation is classified by assigning the label which is most frequent among the $k$ training observations that are nearest to the unlabeled one. Common distances used for identifying the $k$ nearest samples include Euclidean distance for continuous variables, Hamming distance for discrete variables. In this analysis, we used 5-fold cross validated kNN with $k=10$ and Euclidean distance to evaluate the computed phenotypes from k-means clustering.

*3.2 Artificial neural network (ANN)*

An artificial neural network model mimics the biological brain response to multisource stimuli. ANN simulates the brain using a network of interconnected neuron cells to create a massive parallel processor. A neural network is always established with a number of layers, each of which consists several neuron cells. The neuron cells at different layers are connected with specific manners. There are three important components for building a neural network: 1) Training algorithm; 2) Activation function; 3) Network topology. Training algorithm is used to determine the weights $w_{i}$ for the input signals $x_{i}, i=1,\ldots,n$. Activation function $f$ transforms weights and sums inputs to an output $y$.

$$y\left( x \right)=f\left( \sum_{i=1}^{n} w_{i}x_{i} \right)$$

Network topology describes the number of neuron cells, the number of layers and manner in which the cells are connected. In this analysis, we applied 5-fold cross validated ANN with one layer of neuron cells to evaluate the computed phenotypes from k-means clustering.

**4. Dimensionality Reduction**

*4.1 Linear Approaches: Multidimensional scaling (MDS) and Principle component analysis (PCA)*

Multidimensional scaling (MDS) is a method of visualizing the level of similarity of individual cases of a dataset [^6^](#_ENREF_6). An MDS algorithm aims to place each observation in a $N$-dimensional space such that the between-observation distances are preserved as much as possible. Each observation is then assigned coordinates in each of the $N$ dimensions. The number of dimensions of MDS is typically chosen to be 2, which can optimize the object locations for a two-dimensional scatterplot. Principle component analysis (PCA) is another commonly used dimensionality reduction method [^7^](#_ENREF_7). It can be treated as the algorithm of the simplest MDS. The major objective of PCA is to explain the variance in the original signal. It is a mathematical procedure that transforms a number of possibly correlated variables into a smaller number of uncorrelated variables through a process known as orthogonal transformation. PCA minimizes the covariance of the data and yields high-energy orthogonal vectors in terms of the signal variance. It looks for an orthogonal linear transformation that maximizes the variance of the variables.

*4.2 Non-Linear Technique: T-distributed stochastic neighbor embedding (t-SNE)*

T-distributed stochastic neighbor embedding (t-SNE) is a machine learning algorithm for dimensionality reduction [^8^](#_ENREF_8)^,^[^9^](#_ENREF_9). It is a nonlinear dimensionality reduction technique that is particularly well-suited for manifold embedding of very high-dimensional data into 2D or 3D. The t-SNE algorithm comprises two main stages. In the first stage, t-SNE constructs a probability distribution over all pairs of high-dimensional observations in such a way that similar observations have a high probability of being selected, while dissimilar observations have a correspondingly smaller probability of being paired. In the second stage, t-SNE defines a similar probability distribution over the observations in the low-dimensional map, and it minimizes the Kullback-Leibler divergence between the two distributions with respect to the locations of the objects in the map [^10^](#_ENREF_10). In this study, the dimension reduction to 2D and 3D of the 3,297-signature vector of derived neuroimaging biomarkers was performed using both PCA and t-SNE.

### **5. Comparing the distributions of neuroimaging biomarkers across clusters**

Following the k-means and hierarchical clustering of the cases based on the neuroimaging biomarkers, we performed parametric and nonparametric tests to compare the distributions of the biomarkers across different clusters. The parametric Student’s t-test is commonly used to assess differences of sample means when the data approximately follow a normal distribution. The nonparametric Kolmogorov-Smirnov and Mann-Whitney-Wilcoxon tests contrast continuous one-dimensional probability distributions of two samples. Whitney-Wilcoxon test is a nonparametric test of the null hypothesis that it is equally likely that a randomly selected value from one sample will be less than or greater than a randomly selected value from a second sample. It can be used to determine whether two independent samples were selected from populations having the same distribution. Kolmogorov-Smirnov test is also testing whether two samples are drawn from the same distribution. Unlike the Student’s t test, the Kolmogorov-Smirnov and the Whitney-Wilcoxon tests do not require the assumption of normal distribution. The averaged p-values from these three tests were ranked to see which biomarkers are consistently significantly different across the multiple clusters. The conservative Bonferroni correction was applied to control the false-positive rate in these multiple tests. The top twenty significantly different biomarkers were selected as salient neuroimaging features that drive the clustering of the observations. The number of salient markers selected is determined based on the consistency of their significance according to both k-means clustering and hierarchical clustering. All the top twenty selected neuroimaging biomarkers are common in the k-means and the hierarchical clustering, according to the rankings of the significance tests.

### **6. Chi-square test and Fisher’s exact test for the categorical features in the UK Biobank**

Once we obtained the computed phenotypes derived by k-means and hierarchical clustering, we employed Chi-square and Fisher’s exact tests to identify categorical variables in the UK Biobank data that are substantially different between clusters. For two-level categorical variables, the Chi-square test was used, and for categorical features with three or more levels, the Fisher’s exact test was carried out. Again, Bonferroni correction for multiple tests was applied throughout. We used mosaic plots to illustrate the categorical variables that exhibit significantly different distribution patterns across clusters. The magnitudes of the standardized residuals shown in the mosaic plots illustrate the significance of the testing differences.

### **7. Decision trees and random forests**

*7.1 Decision tree*

Decision tree is a widely used algorithm of supervised learning method. It is mostly used in classification, but can also be applied in regression [^11^](#_ENREF_11)^,^[^12^](#_ENREF_12). Decision trees split the population, or entire sample, into two or more homogeneous sets or sub-populations based on most significant splitter among all the input variables. The advantage of decision trees is the induced clear interpretation and illustration of the protocol. Since they represent non-parametric methods for classification and regression, there are no assumptions about the distribution and the classifier structure. Over fitting is one of the most practical difficulty for using decision trees.

*7.2 Random forest*

Random forest is an ensemble learning method developed by Leo Breiman for classification, regression and other tasks [^13^](#_ENREF_13). It works by fitting a large number of decision trees (a forest) and outputs class labels that represent the mode of the classes given by the individual trees in the application of classification. Alternatively, the mean of the prediction of the individual trees in the application of regression may be reported as the outcome class label. Random forest overcomes the overfitting problem of decision trees. Different from fitting a single decision tree, which may have large variance or large bias, random forests use the strategy of *averaging* to find a balance between the two extremes, trading off bias and precision. Each of the decision trees generated by random forest is built using a bootstrap sample of the data. A random forest uses both bagging (bootstrap aggregation) and random variable selection for tree building, where at each split, the splitting variable is selected from a random set of candidate variables. The variable importance is determined by various measures like the mean decrease Gini value. Gini importance measures the average gain of purity by splitting a variable. Permuting a useful variable is likely to give relatively large decrease in mean Gini gain. Therefore, the ranking based on mean decrease Gini value reflects the importance of the variables in classifying the data.

The salient neuroimaging biomarkers and the significant categorical variables were analyzed by random forest. In each trial of the random forest algorithm, one categorical variable related to mental disorder was classified using all the other variables. Five-fold cross validation was repeated 10 times to assess the accuracy, reliability, sensitivity, and specificity of the iterative prediction.

## **Supplementary Results**

**Figure S2** shows a plot of the number of clusters (x-axis) vs. the total within sum of squares (y-axis), which is used to determine the optimal number of clusters. The within sum of squares results shown in **Figure S2**, paired with the silhouette values shown in **Figure 1** in the main text, suggest that an optimal number of clusters between 2 and 4. We chose 2 clusters because they give the highest overall silhouette values across clusters (see **Figure 1**).


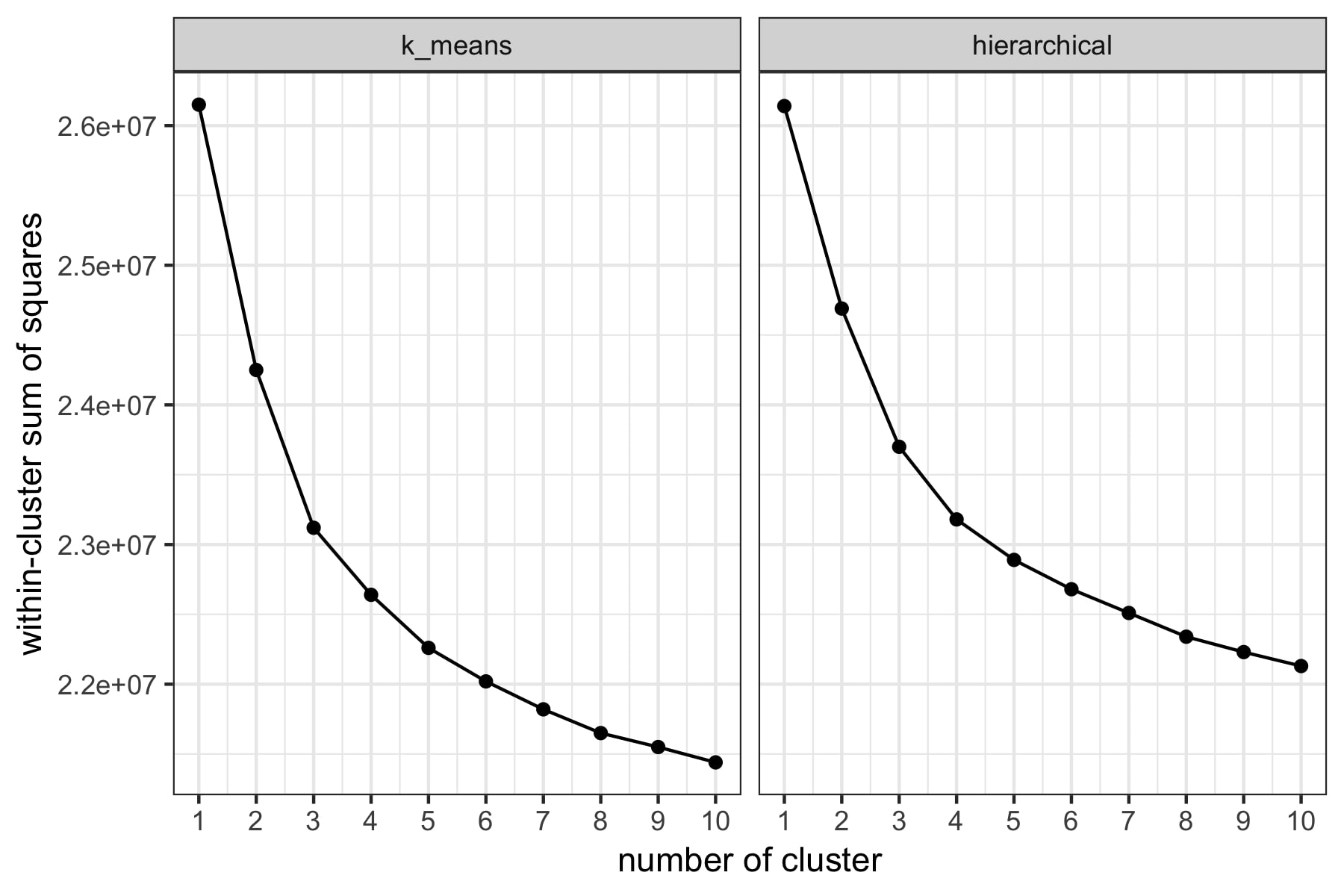


**Figure S2.** Clustering optimization based on total within-cluster sum of squares for (a) k-means clustering (b) hierarchical clustering.

**Table S1**. Consistency of k-means clustering based on 1,000 repeated clustering experiments with random initialization.

| **Cluster** | **Consistency** | **Variance** | **Cluster-size** | **Silhouette** |
| --- | --- | --- | --- | --- |
| 1 | 0.9984 | 0.0094 | 4,242 | 0.09 |
| 2 | 0.9990 | 0.0096 | 3,689 | 0.05 |

**Table S2**. Contingency table showing the concordance and discordance of clustering between k-means clustering (columns) and hierarchical clustering (rows).

|  | | **k-means clustering** | |
| --- | --- | --- | --- |
| **hierarchical clustering** |  | **Cluster 1** | **Cluster 2** |
|  | **Cluster 1** | 3,429 (43.2%) | 813 (10.3%) |
|  | **Cluster 2** | 652 (8.2%) | 3,037 (38.3%) |

The optimal linkage criterion used in hierarchical clustering is determined according to the agglomerative coefficient. The agglomerative coefficient values for different linkage criteria are shown in **Table S3**, which indicates that Ward’s linkage should be used since it gives the maximum agglomerative coefficient values as 0.963.

**Table S3.** Agglomerative coefficient values with different linkage criteria for hierarchical clustering.

| **Linkage Criterion** | **Agglomerative Coefficient** |
| --- | --- |
| Average | 0.779 |
| Single | 0.781 |
| Complete | 0.833 |
| Ward’s | 0.963 |

**Table S4**. Summary statistics for the significantly different categorical features detected by Chi-square and Fisher’s exact tests between the two computed phenotypes.

| **Variable** | **Cluster 1** | **Cluster 2** | **Significance** |
| --- | --- | --- | --- |
| **Sex**  Female  Male | 3,239 (76.4%)  1,003 (23.6%) | 909 (24.6%)  2,780 (75.4%) | $***$ |
| **Sensitivity/hurt feelings**  No  Yes | 1,698 (40.0%)  2,429 (57.3%) | 1,890 (51.2%)  1,707 (46.3%) | $***$ |
| **Worrier/anxious feelings**  No  Yes | 1,748 (41.2%)  2,407 (56.7%) | 1,879 (50.9%)  1,750 (47.4%) | $***$ |
| **Risk taking**  No  Yes | 3,148 (74.2%)  915 (21.6%) | 2,463 (66.8%)  1,101 (29.8%) | $***$ |
| **Guilty feelings**  No  Yes | 2,797 (65.9%)  1,377 (32.5%) | 2,736 (74.2%)  895 (24.3%) | $***$ |
| **Seen doctor for nerves, anxiety, tension or depression**  No  Yes | 2,647 (62.4%)  1,575 (37.1%) | 2,595 (70.3%)  1,081 (29.3%) | $***$ |
| **Alcohol usually taken with meals**  No  Yes | 608 (14.3%)  2,015 (47.5%) | 7,51 (20.4%)  1,500 (40.7%) | $***$ |
| **Snoring**  No  Yes | 2,647 (62.4%)  1,315 (31.0%) | 2,083 (56.5%)  1,425 (38.6%) | $***$ |
| **Worry too long after embarrassment**  No  Yes | 1,936 (45.6%)  2,156 (50.8%) | 1,989 (53.9%)  1,602 (43.4%) | $***$ |
| **Miserableness**  Yes  No | 2,279 (53.7%)  1,904 (44.9%) | 2,264 (61.4%)  1,389 (37.7%) | $***$ |
| **Ever highly irritable/argumentative for 2 days**  No  Yes | 3,524 (83.1%)  593 (14.0%) | 3,241 (87.9%)  395 (10.7%) | $*$ |
| **Nervous feelings**  Yes  No | 3,192 (75.2%)  941 (22.2%) | 2,969 (80.5%)  659 (17.9%) | $**$ |
| **Ever depressed for a whole week**  No  Yes | 1,925 (45.4%)  2,203 (51.9%) | 1,876 (50.9%)  1,756 (47.6%) | $*$ |
| **Ever unenthusiastic/disinterested for a whole week**  No  Yes | 2,645 (62.4%)  1,410 (33.2%) | 2,481 (67.3%)  1,093 (29.6%) | $*$ |
| **Sleepless/insomnia**  Never/rarely  Sometimes  Usually | 927 (21.9%)  2,060 (48.6%)  1,251 (29.5%) | 1,095 (29.7%)  1,765 (47.8%)  827 (22.4%) | $***$ |
| **Getting up in morning**  Not at all easy  Not very easy  Fairly easy  Very easy | 198 (4.6%)  661 (15.6%)  2,143 (50.5%)  1,180 (27.8%) | 113 (3.1%)  440 (11.9%)  1,835 (49.7%)  1,249 (33.9%) | $***$ |
| **Nap during day**  Never/rarely  Sometimes  Usually | 2,571 (60.6%)  1,450 (34.2%)  177 (4.2%) | 2,014 (54.6%)  1,413 (38.3%)  249 (6.7%) | $***$ |
| **Frequency of tiredness/lethargy in last 2 weeks**  Not at all  Several days  More than half the days  Nearly everyday | 1,971 (46.5%)  1,708 (40.3%)  234 (5.5%)  234 (5.5%) | 1,939 (52.6%)  1,405 (38.1%)  153 (4.1%)  154 (3.9%) | $***$ |
| **Alcohol drinker status**  Never  Previous  Current | 145 (3.4%)  114 (2.7%)  3,981 (93.8%) | 60 (1.6%)  70 (1.9%)  3,557 (96.4%) | $***$ |

Significance code: *** p-value $<1\times{10}^{-8}$; ** p-value $<1\times{10}^{-6}$; * p-value $<1\times{10}^{-4}$.

**
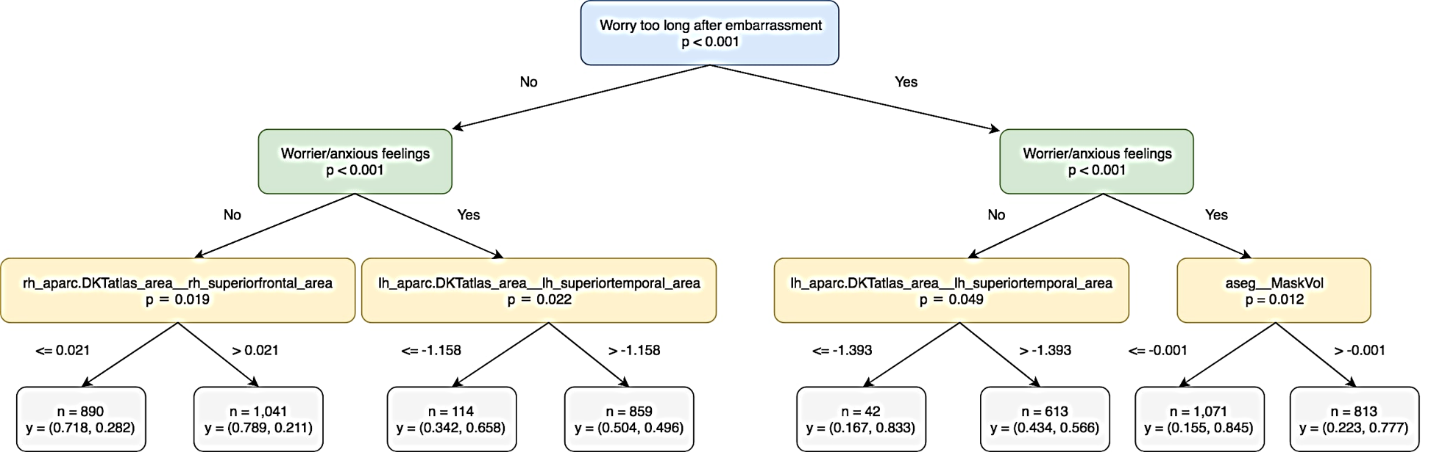
**

**Figure S3:** An example of a decision tree illustrating a simple clinical decision support system that can provide machine guidance for identifying patients with sensitivity/hurt feelings based on categorical variables and neuroimaging biomarkers. In each terminal node, the y vector includes the percentage of subjects being split into the downstream nodes or branches, in this case, answering “yes“ or “no” to the question whether having “*Sensitivity/hurt feelings*.” The p-values listed at branching nodes indicate the significance of the corresponding splitting criterion.

**Figure S4** illustrates the distributions of the scaled twenty biomarkers in the middle of the feature importance ranking and at the bottom of the ranking based on parametric and non-parametric tests according to k-means clustering. As expected, the distributions overlapped more compared to the top twenty biomarkers selected in the main text.


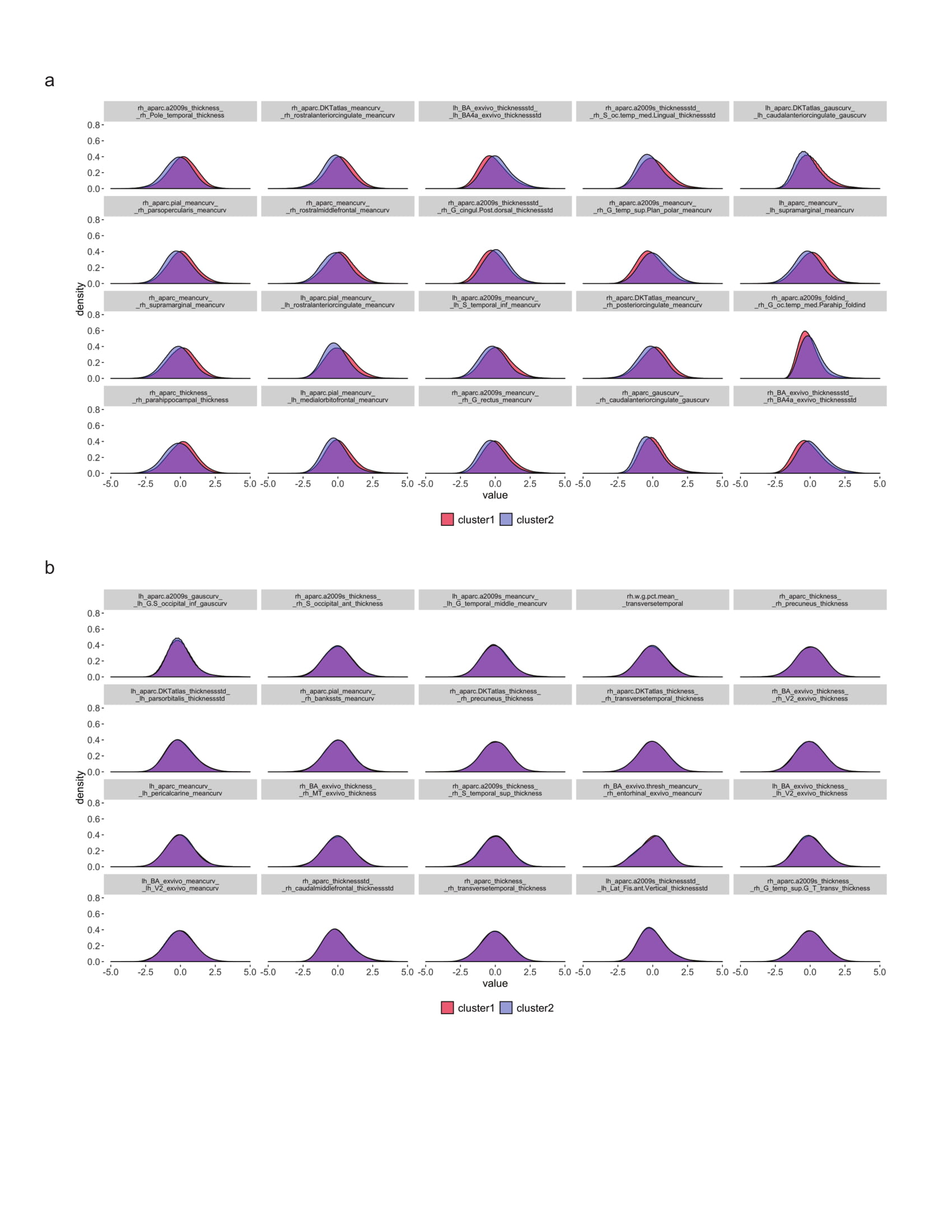


**Figure S4.** Density plots for the scaled (a) twenty biomarkers in the middle of the feature importance ranking; (b) twenty biomarkers at the bottom of the ranking of the significantly different brain neuroimaging biomarkers based on k-means clustering.

The non-linear t-SNE manifold projection enables deeper computable phenotype investigations. **Figure S5** shows one example of stratifying a smaller cohort of participants (n=86) off from cluster 2. **Table S5** shows the correspondent sample sizes of the three clusters.

**
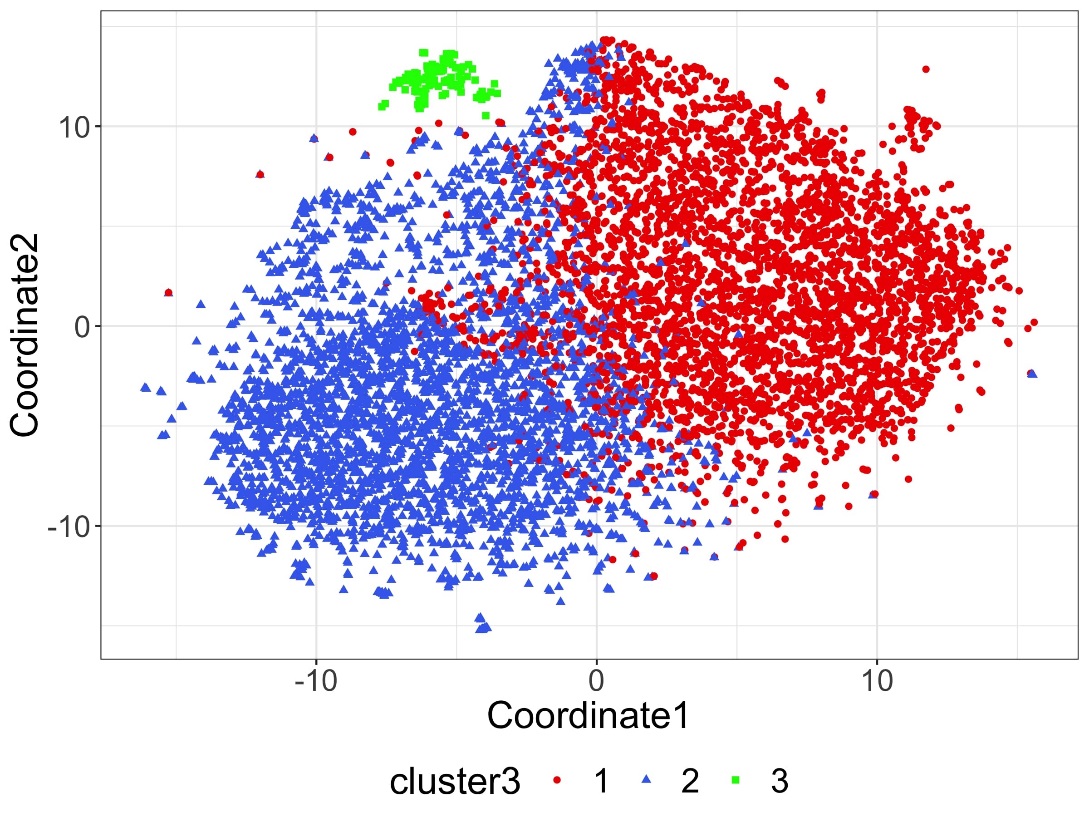
**

**Figure S5.** 2-dimensional t-SNE of the brain neuroimaging biomarkers with clustering label 1 and 2 generated by k-means clustering. The small sub-cluster separated from the major cluster 2 is indicated in green as cluster 3.

**Table S5.** Number of subjects in each cluster.

|  | **cluster 1 (red)** | **cluster 2 (blue)** | **cluster 3 (green)** |
| --- | --- | --- | --- |
| Sample size | 4,143 | 3,660 | 128 |

Distributions of the selected twenty salient neuroimaging biomarkers in cluster 1, 2 and 3 are illustrated in **Figure S6a.** Since the majority of the small cluster 3 was initially part of cluster 1, not surprisingly nineteen out of twenty biomarker distributions in cluster 3 are almost overlapping with the correspondent distributions in cluster 1. However, the t-SNE plot suggests a possible separation of clusters 2 and 3. We have a total of ~1,000 categorical features in the UKBB dataset, with ~38% (384) with 2, 3 or 4 categories each. The remaining ~62% included 5 to 16 categories. We focused our analysis on the 384 features and found no significant differences between cluster 2 and 3 (data not shown). We then looked at the neuroimaging biomarkers retrieved by the MRI data to see if any was significant enough to separate these two clusters. We performed three tests to compare the distributions of all the neuroimaging biomarkers across cluster 2 and 3, namely one parametric test (i.e., t test), and two nonparametric (i.e., Kolmogorov-Smirnov and Whitney-Wilcoxon, the latter shown in **Table S6**).

**Figure S6b** shows the distributions of ten neuroimaging biomarkers that are significantly different between clusters 2 and 3, while the summary statistics of their unscaled values are shown in **Table S6**. All biomarkers are associated to pre-central and post-central gyrus cortical tissue areas in the right hemisphere of the brain. Even if the 2 groups are largely unbalanced (128/green vs 3,660/blue patients, as shown in **Table S5**), we investigated the possibility that a latent characteristic (not measured in the UKBB dataset) can be inferred by performing a meta-analysis in the literature of the ten brain neuroimaging biomarkers listed in **Table S6**.


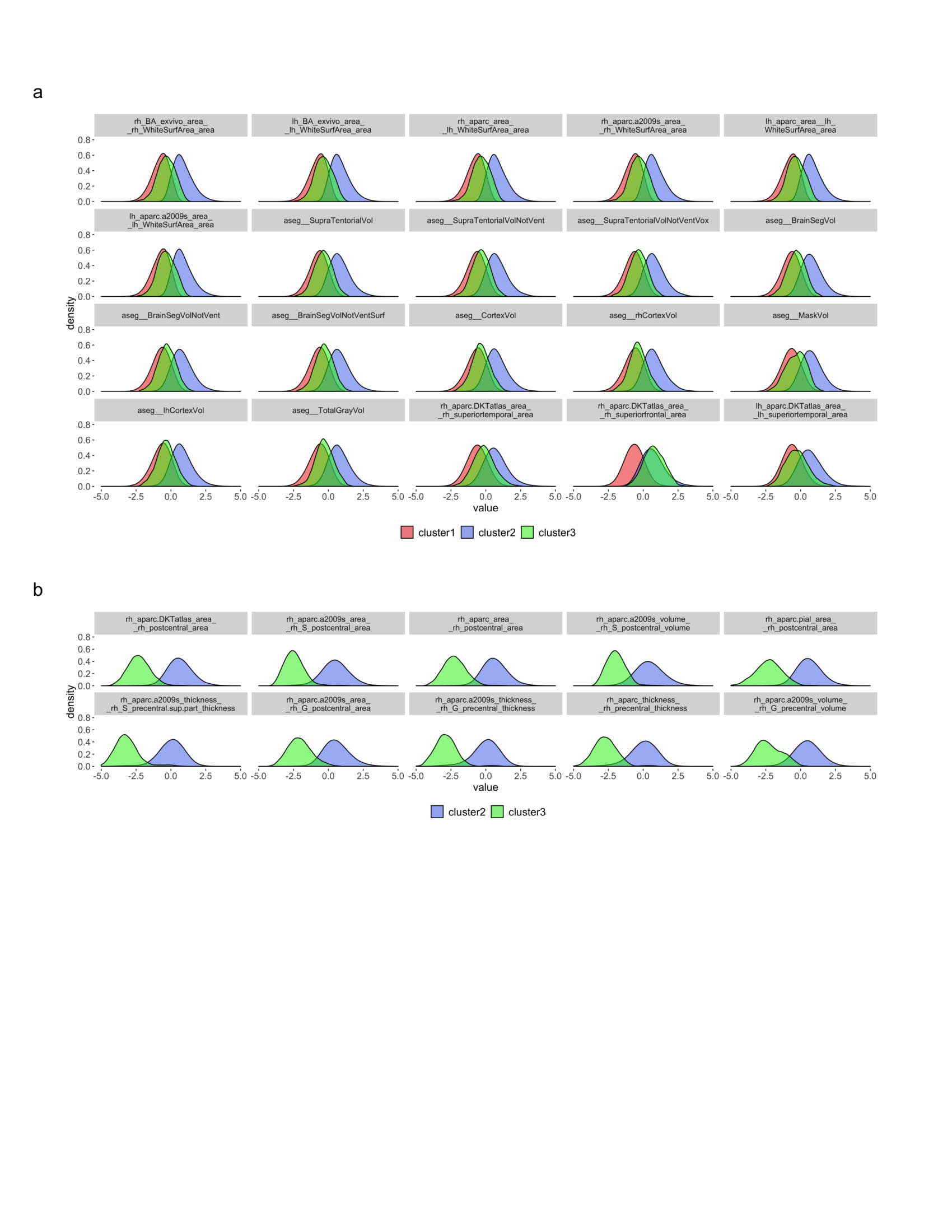


**Figure S6.** (a) Density plots of the scaled top twenty selected brain neuroimaging biomarkers by k-means clustering. The green cluster is the sub-cluster identified by t-SNE. (b) Density plots for the scaled brain neuroimaging biomarkers related to precentral and postcentral area represent the highly discriminating feature distributions separating clusters 2 and 3 based on k-means clustering.

**Figure S6b** and **Table S6** show that the pre and post central gyrus cortical surface area biomarkers are consistently and significantly smaller for cluster 3 (green). These neuroimaging biomarkers with reduced values (again, all localized in the right hemisphere) might indicate reduced functional activities, as many studies have shown. For instance, Kuperberg and colleagues demonstrated selective thinning of the cerebral prefrontal cortices (including precentral and postcentral gyri) in patients with schizophrenia [^14^](#_ENREF_14). Others have shown similar reductions of pre- and post-central areas in bipolar disease and Williams syndrome ^[15-17](#_ENREF_15)^.

|  | **cluster 2** | | | **cluster 3** | | |  |
| --- | --- | --- | --- | --- | --- | --- | --- |
| **Name** | **Mean** | **Median** | **SD** | **Mean** | **Median** | **SD** |  |
| Post-central S cortical surface area of the in the right Hemisphere (in DKTatlas)  (*rh_aparc.DKTatlas_area__rh_postcentral_area*) | 4687 | 4653 | 451 | 3107 | 3098 | 388 | $***$ |
| rh_aparc.a2009s_area__rh_S_postcentral_area | 1919 | 1911 | 286 | 1009 | 978 | 197 | $***$ |
| Post-central cortical surface area in the right hemisphere (*rh_aparc_area__rh_postcentral_area*) | 4229 | 4197 | 409 | 2830 | 2822 | 364 | $***$ |
| Volume of the post-central S in the right hemisphere  (rh_aparc.a2009s_volume__rh_S_postcentral_volume) | 3596 | 3569 | 604 | 2103 | 2036 | 363 | $***$ |
| Post-central cortical surface area of the right hemisphere (rh_aparc.pial_area__rh_postcentral_area) | 5128 | 5101 | 510 | 3401 | 3412 | 534 | $***$ |
| Thickness of the pre-central S superior part in the RH  (*rh_aparc.a2009s_thickness__rh_S_precentral.sup.part_thickness*) | 2.34 | 2.35 | 0.19 | 1.67 | 1.66 | 0.17 | $***$ |
| rh_aparc.a2009s_area__rh_G_postcentral_area | 1402 | 1386 | 175 | 893 | 876 | 148 | $***$ |
| Thickness of pre-central G in the right hemisphere  (*rh_aparc.a2009s_thickness__rh_G_precentral_thickness*) | 2.74 | 2.76 | 0.23 | 2.03 | 2.02 | 0.18 | $***$ |
| Thickness of pre-central area in the right hemisphere  (*rh_aparc_thickness__rh_precentral_thickness*) | 2.50 | 2.51 | 0.17 | 2.02 | 2.00 | 0.14 | $***$ |
| Volume of the pre-central G in the right hemisphere (rh_aparc.a2009s_volume__rh_G_precentral_volume) | 6258 | 6251 | 844 | 3664 | 3516 | 835 | $***$ |

**Table S6.** Summary statistics of the unscaled values for the brain neuroimaging biomarkers related to precentral and postcentral area that are significantly differently distributed between cluster 2 and 3 based on parametric and non-parametric tests. Significance code: “***” p-value $<1\times{10}^{-8}$. The p-values are generated from Whitney-Wilcoxon tests. Legend: RH=right hemisphere, S=sulcus, G=g­yrus, sup.part=superior part.

**References**

1 Almeida, J. S. & Prieto, C. A. Automated unsupervised classification of the Sloan Digital Sky Survey stellar spectra using k-means clustering. *The Astrophysical Journal* **763**, 50 (2013).

2 Aggarwal, C. C. & Reddy, C. K. *Data clustering: algorithms and applications*. (CRC Press, 2013).

3 Filzmoser, P., Baumgartner, R. & Moser, E. A hierarchical clustering method for analyzing functional MR images. *Magnetic Resonance Imaging* **17**, 817-826, doi:<http://dx.doi.org/10.1016/S0730-725X(99)00014-4> (1999).

4 Mirkin, B. in *Classification, data analysis, and data highways* 172-181 (Springer, 1998).

5 Murtagh, F. A survey of recent advances in hierarchical clustering algorithms. *The Computer Journal* **26**, 354-359 (1983).

6 Steyvers, M. Multidimensional scaling. *Encyclopedia of cognitive science* (2002).

7 Jolliffe, I. *Principal component analysis*. (Wiley Online Library, 2002).

8 Van Der Maaten, L. Accelerating t-SNE using tree-based algorithms. *Journal of machine learning research* **15**, 3221-3245 (2014).

9 Maaten, L. v. d. & Hinton, G. Visualizing data using t-SNE. *Journal of machine learning research* **9**, 2579-2605 (2008).

10 Van Erven, T. & Harremos, P. Rényi divergence and Kullback-Leibler divergence. *IEEE Transactions on Information Theory* **60**, 3797-3820 (2014).

11 Twala, B., Jones, M. & Hand, D. J. Good methods for coping with missing data in decision trees. *Pattern Recognition Letters* **29**, 950-956 (2008).

12 Dinov, I. *Data Science and Predictive Analytics: Biomedical and Health Applications using R*. <http://Predictive.Space> (Springer International Publishing, 2018).

13 Breiman, L. Random forests. *Machine learning* **45**, 5-32 (2001).

14 Kuperberg, G. R. *et al.* Regionally localized thinning of the cerebral cortex in schizophrenia. *Archives of general psychiatry* **60**, 878-888 (2003).

15 Lemaitre, H. *et al.* Normal age-related brain morphometric changes: nonuniformity across cortical thickness, surface area and gray matter volume? *Neurobiology of aging* **33**, 617. e611-617. e619 (2012).

16 Rimol, L. M. *et al.* Cortical volume, surface area, and thickness in schizophrenia and bipolar disorder. *Biological psychiatry* **71**, 552-560 (2012).

17 Thompson, P. M. *et al.* Abnormal cortical complexity and thickness profiles mapped in Williams syndrome. *Journal of Neuroscience* **25**, 4146-4158 (2005).

# **Acknowledgments**

Partially support for this study was provided by NIH grants P20 NR015331, P50 NS091856, P30 DK089503, U54 EB020406, UL1TR002240, and P30AG053760, NSF grants 1734853, 1636840, 1416953, 0716055 and 1023115, and the Elsie Andresen Fiske Research Fund. Many colleagues from the Statistics Online Computational Resource (SOCR), Center for Complexity and Self-management of Chronic Disease (CSCD), Big Data Discovery Science (BDDS), and the Michigan Institute for Data Science (MIDAS) provided comments and suggestions. This study was made possible and conducted under UK Biobank application number 25641.

**Author Contributions**

YZ: developed methods, implemented computational algorithms, performed data analytics, wrote the manuscript, interpreted results

LZ: retrieved and processed the data, wrote the manuscript

NZ: developed methods, wrote the manuscript

YZ: developed methods, wrote the manuscript

SM: developed methods, wrote the manuscript, interpreted results

TW: developed methods, wrote the manuscript

HS: developed methods, wrote the manuscript

AT: conceptualized the study, wrote manuscript

ID: conceptualized the study, developed methods, performed data analytics, wrote the manuscript, interpreted results
